# Association between continuous hyperosmolar therapy and survival in patients with traumatic brain injury – a multicentre prospective cohort study and systematic review

**DOI:** 10.1186/s13054-017-1918-4

**Published:** 2017-12-28

**Authors:** Karim Asehnoune, Sigismond Lasocki, Philippe Seguin, Thomas Geeraerts, Pierre François Perrigault, Claire Dahyot-Fizelier, Catherine Paugam Burtz, Fabrice Cook, Dominique Demeure dit latte, Raphael Cinotti, Pierre Joachim Mahe, Camille Fortuit, Romain Pirracchio, Fanny Feuillet, Véronique Sébille, Antoine Roquilly, For the ATLANREA group, Nicolas Nesseler, Yoann Launey, Olivier Mimoz, Thomas Kerforne, Martine Ferrandière, Francis Remerand, Pascale le Maguet, Yannick Malledant, For the COBI group, Mathieu Martin, Russel Chabanne, Laurent Muller, Jean Luc Hanouz, Jeremy Allary, Hervé Floch, Claire Roger, Anne-Claire Lukaszewicz, Matthieu Biais, Perrine Boucheix, Soizic Gergaud, Benoit Plaud, Jean Michel Constantin, Fouad Marhar

**Affiliations:** 1 Intensive Care Unit, Anaesthesia and Critical Care Department, Hôtel Dieu - HME, CHU Nantes, Nantes, France; 2 Department of Anaesthesiology and Critical Care Department, University Hospital of Angers, Angers, France; 3 Intensive Care Unit, Anaesthesia and Critical Care Department, Pontchaillou, University Hospital of Rennes, Rennes, France; 4 Anaesthesia and Critical Care Department, University Hospital of Toulouse, Toulouse, France; 5 Intensive Care Unit, Anaesthesia and Critical Care Department, Gui Chauliac University Hospital of Montpellier, Montpellier, France; 6 Neuro-Intensive Care Unit, Anaesthesia and Critical Care Department, Poitiers, University Hospital of Poitiers, Poitiers, France; 7 Intensive Care Unit, Anaesthesia and Critical Care Department, Beaujon, University Hospital of Beaujon (AP-HP), Beaujon, France; 8 Intensive Care Unit, Anaesthesia and Critical Care Department, Henri Mondor, University Hospital of Créteil (AP-HP), Créteil, France; 9 Department of Anesthesia and Critical care Medicine, Hôpital Européen Georges Pompidou, Paris 5 Descartes, Sorbonne Paris Cité, Paris, France; 10 NSERM UMR-S1153, Team ECSTRA, Hôpital Saint Louis, Paris, France; 11 UMR 1246 SPHERE “methodS in Patients-centered outcomes and HEalth ResEarch”, Nantes University, Nantes, France; 12 Plateforme de Biométrie, Département Promotion de la Recherche Clinique, University Hospital of Nantes, Nantes, France; 13 CHU de Nantes, Service d’Anesthésie Réanimation, 1 place Alexis Ricordeau, 44093 Nantes, Cedex 1, France

**Keywords:** Trauma, Traumatic brain injury, Intracranial hypertension, Brain oedema, Hyperosmolar therapy, Saline solution, Hypertonic, Sodium

## Abstract

**Background:**

Intracranial hypertension (ICH) is a major cause of death after traumatic brain injury (TBI). Continuous hyperosmolar therapy (CHT) has been proposed for the treatment of ICH, but its effectiveness is controversial. We compared the mortality and outcomes in patients with TBI with ICH treated or not with CHT.

**Methods:**

We included patients with TBI (Glasgow Coma Scale ≤ 12 and trauma-associated lesion on brain computed tomography (CT) scan) from the databases of the prospective multicentre trials Corti-TC, BI-VILI and ATLANREA. CHT consisted of an intravenous infusion of NaCl 20% for 24 hours or more. The primary outcome was the risk of survival at day 90, adjusted for predefined covariates and baseline differences, allowing us to reduce the bias resulting from confounding factors in observational studies. A systematic review was conducted including studies published from 1966 to December 2016.

**Results:**

Among the 1086 included patients, 545 (51.7%) developed ICH (143 treated and 402 not treated with CHT). In patients with ICH, the relative risk of survival at day 90 with CHT was 1.43 (95% CI, 0.99–2.06, *p* = 0.05). The adjusted hazard ratio for survival was 1.74 (95% CI, 1.36–2.23, *p* < 0.001) in propensity-score-adjusted analysis. At day 90, favourable outcomes (Glasgow Outcome Scale 4–5) occurred in 45.2% of treated patients with ICH and in 35.8% of patients with ICH not treated with CHT (*p* = 0.06). A review of the literature including 1304 patients from eight studies suggests that CHT is associated with a reduction of in-ICU mortality (intervention, 112/474 deaths (23.6%) vs. control, 244/781 deaths (31.2%); OR 1.42 (95% CI, 1.04–1.95), *p* = 0.03, *I*_2_ = 15%).

**Conclusions:**

CHT for the treatment of posttraumatic ICH was associated with improved adjusted 90-day survival. This result was strengthened by a review of the literature.

**Electronic supplementary material:**

The online version of this article (doi:10.1186/s13054-017-1918-4) contains supplementary material, which is available to authorized users.

## Background

Severe trauma is responsible of more than 5 million deaths every year worldwide and this incidence is expected to increase in the coming decades [1]. Traumatic brain injury (TBI) is the most severe condition observed in trauma patients, given that nearly 33% of patients with TBI die in hospital and another 33% have poor neurological recovery [2]. The sequelae and changes in quality of life observed after severe TBI are associated with an excess risk of death long after hospital discharge [3]. Therapies are therefore urgently needed to decrease mortality and the tremendous medical costs of TBI [4].

Prevention and treatment of intra-cranial hypertension (ICH) are the cornerstones of treatment for patients with TBI in intensive care units (ICUs), as uncontrolled ICH worsens brain damage and remains the most common cause of death after severe TBI [5]. Several strategies are recommended for the treatment of ICH but few have been demonstrated to improve long-term outcomes [6]. After sedation and head positioning, boluses of hyperosmolar therapy are frequently the second-tier strategy of ICH treatment, but the induced reduction in intracranial pressure (ICP) is transient and a rebound of ICH is frequently observed after a few hours [7, 8]. Continuous infusion of hyperosmolar therapy has therefore been proposed for the treatment of patients with severe brain injury, but its effects on survival and outcomes have been disappointing [9].

A recent retrospective study performed at our institution suggested that ICP was better controlled in patients with TBI with ICH refractory to barbiturates when using continuous hyperosmolar therapy [10]. We therefore aimed to investigate the effects of early administration of continuous hyperosmolar therapy in patients with TBI with ICH on mortality and long-term outcomes. We used data collected in three prospective trials involving patients with TBI to compare mortality (primary objective) and long-term outcomes (secondary objective) in patients with TBI with ICH treated or not with early continuous hyperosmolar therapy (CHT). Given the observational design of the study, we planned a priori to use a propensity adjustment for the comparison of the primary outcome between treated and untreated patients because it enabled us to reduce the bias resulting from confounding factors in observational studies. Finally, we conducted a systematic review incorporating the findings of this trial.

## Methods

### Ethics approval

The study protocol of this sub-study of three prospective trials was approved by an institutional review board (Comité d'Éthique de la Recherche en Anesthésie-Réanimation, CERAR#2016-01-03). Informed consent was waived owing to the non-interventional design of this study and because relatives and patients provided informed consent for the collection of the medical data in the original studies.

### Study design

Prospectively collected individual patient data from three studies were pooled together. The CORTI-TC trial was a multicentre (19 ICUs), randomised, double-blind, placebo-controlled trial of hydrocortisone and fludrocortisone in patients with TBI (NCT01093261) [11]. The BI-VILI study (NCT01885507) was a multicentre (20 ICUs) before-after evaluation of a quality improvement project, aiming to promote protective mechanical ventilation (association of low tidal volume and moderate positive end-expiratory pressure) in brain-injured patients [12]. The ATLANREA cohort (NCT02426255) was a prospective non-interventional multicentre (four ICUs) follow up of brain-injured patients requiring more than 24 hours of mechanical ventilation [13]. All the centres are experts in the care of patients with TBI and each centre receive more than 50 severe patients with TBI per year.

### Population

We included all patients with TBI from 15 to 75 years of age with a duration of mechanical ventilation ≥ 24 hours. Inclusion criteria were patients with moderate (Glasgow Coma Scale (GCS) score 9–12) to severe (GCS score 3–8) traumatic brain injury with one or more acute processes related to trauma on cerebral computed tomography (CT) imaging. Exclusion criteria were non-traumatic brain injury or an early decision to withdraw care (during the first 24 hours in the ICU).

### Definition of intra-cranial hypertension

ICH was defined as one or more in-ICU episodes of ICP higher than 20 mmHg [6] with a duration that required specific therapeutic intervention.

### General care of brain-injured patients (stage 1 treatment)

Investigators followed the brain trauma foundation guidelines for TBI resuscitation [6], except for one centre, which used CHT as a first-line treatment for ICH. All patients were sedated with a continuous intravenous infusion of hypnotic and morphinic agents and were mechanically ventilated. Sedated patients were kept in a semi-recumbent position unless there were contraindications to this. Secondary brain injuries were prevented by keeping body temperature between 36.0 °C and 37.0 °C, ensuring normoglycaemia and normocapnia and avoiding hypoxaemia (stage 1 treatment, Additional file 1: Figure S1). Natraemia was tested in blood twice a day in the control group (or more frequently in the case of abnormalities), and normal natraemia (138–145 mmol/L) was maintained in the absence of ICH (Additional file 1: Figure S1). Intracranial pressure was monitored with an intra-parenchymal probe (Codman, Johnson and Johnson Company, Raynham, MA, USA.) placed in the most affected side of the brain as identified on CT. External-ventricular drainage was performed in the case of hydrocephalus.

### Treatment of intracranial hypertension (stage 2 and 3 treatments)

A bolus of hyperosmolar therapy (mannitol 0.25 to 1 g/kg body weight [6] or hypertonic saline solution, 250 mOsm dose [7]) was routinely used as first-line treatment to control episodes of ICH. Boluses of hyperosmolar therapy were repeated in the case of poor ICP control (ICP > 20 mmHg) and when plasma osmolality remained < 320 mosm/L. When control of ICH was poor despite optimized hyperosmolar therapy (ICP > 20 mmHg), barbiturate (sodium thiopental with a loading dose of 2–3 mg.kg^-1^ followed by a continuous infusion of 2–3 mg.kg^-1^.h^-1^), moderate hypothermia (33–35 °C), moderate hypocapnia (partial arterial pressure of carbon dioxide (PaCO_2_) 32–36 mmHg) and decompressive craniectomy were used according to each centre's protocols and following international recommendations [6]. The control group received stage 1 and 2 treatments but without continuous hyperosmolar therapy (Additional file 1: Figure S1).

### Early continuous hypertonic saline therapy

In one of the participating centres, continuous hyperosmolar saline therapy was infused as the first-line treatment of intracranial hypertension (i.e. when stage 1 treatments had failed). Continuous hypertonic saline therapy consisted of a 1-hour bolus of hypertonic saline solution (20% hypertonic saline solution) followed by an intravenous infusion for a duration of 24 hours or more, prolonged as long as required to control ICP. Continuous hyperosmolar therapy was routinely adapted to the blood level of sodium measured before the first bolus, then every 4–8 hours during the treatment. As previously described (Additional file 2: Figure S2 and [10]), the attending physician set an increasing target of natraemia that could be increased by increments of 5 mmol/L (up to 155 mmol/L) according to the evolution of ICP. In the case of poor control of ICP, second-step treatments were administered. For treatment discontinuation, the target natraemia was gradually decreased to 145 mmol/L (by decrements of 5 mmol/L).

### Data handling

For each of the included studies, data were collected prospectively using the specific websites of each trial. Detailed information explaining instructions for data collection and definitions for outcomes were made available to all investigators before data collection started. For quality assurance purposes, data were electronically checked for uniformity and completeness. Errors or unfilled fields generated queries that were returned to each centre for correction. Missing data are described in the “Results” section.

### Endpoints

The primary endpoint was the risk of survival at day 90 in patients receiving or not receiving CHT for the treatment of ICH. Because we anticipated imbalances in key risk factors at baseline among patients developing ICH treated or not with CHT, the primary outcome was adjusted for such imbalances. We also calculated the crude mortality at day 90.

The secondary endpoint was the dichotomized Glasgow Outcome Scale (GOS) at day 90 (GOS 1–3 vs. 4–5). Safety was investigated through the time course of the blood level of sodium, urea and creatinine in the first 5 days of therapy and the rate of central pontine myelinolysis.

### Statistical analysis

First, in order to identify baseline differences associated with CHT, univariate analysis was applied using the chi-square test for categorical data, Student's *t* test or Wilcoxon test was used for continuous data and the log-rank test for censored data.

For primary analysis, as we previously described [14], propensity score analysis (based on inverse probability weighting) was applied to estimated 90-day survival. The propensity score included predefined covariates (CT classification, age, GCS, non-reactive pupil, hypoxaemia and interventional study arms) [15] and covariates identified by univariate analysis (Cox models were estimated; if *p* values were < 0.20 then the variable was selected, then a backward selection procedure was applied to keep only variables that were significant at the 5% level).

As for sensitivity analysis, we also calculated the non-adjusted hazard ratio (univariate Cox model) and we produced a multivariate Cox model (adjusted on covariates included in the propensity score). The proportional hazards assumption was inspected using Schoenfeld residuals.

Continuous data were expressed as mean ± standard deviation for parametric data and as median (25^th^ to 75^th^ percentiles) for non-parametric data. Categorical data were expressed as number and percentage. A two-sided *p* value <0.05 was considered statistically significant. Statistical analysis was performed with SAS statistical software (SAS 9.3 Institute, Cary, NC, USA).

### Systematic review

Meta-analyses and systematic reviews of observational studies (MOOSE) guidelines were followed in the design and implementation of this systematic review of the literature. We attempted to identify all relevant studies published in English regardless of publication status (published or in press). We considered abstracts presented at scientific meetings < 3 years ago (Society of Critical Care Medicine, European Society of Intensive Care Medicine, Societe Française d’Anesthesie-Reanimation, Societe de Reanimation de Langue Française). PubMed® (MEDLINE/Index Medicus) and the Cochrane Controlled Trials Register were searched for studies published from January 1969 until 31 December 2016. The Medical Subject Heading terms used for the search were “Intracranial Hypertension/drug therapy” OR “Sodium Lactate/therapeutic use” OR “Brain Edema/drug therapy” AND “Saline Solution, Hypertonic/administration & dosage” with the limit “human”. The “related articles” hyperlinks in Medline were explored for additional references. The reference lists of all selected trials and previously published meta-analyses were checked for additional references. The authors selected all studies that evaluated CHT in brain-injured patients. We selected the following key outcomes: number of in-ICU deaths, rate of ICH and rate of severe hypernatraemia (Na^+^ > 160 mmol/L). Treatment effects were reported as RRs with 95% confidence intervals for discontinuous outcomes. Analyses were performed using RevMan® version 5.3 using fixed-effects models with random-effects models for comparison (see Additional file 3: supplemental methods).

## Results

**Fig. 1:**
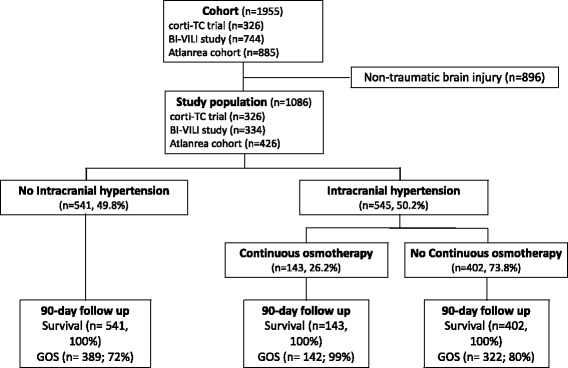
Flow chart. *GOS* Glasgow Outcome Scale

Among the 1086 included patients, 545 (50.2%) developed ICH, among whom 143 (26.2%) received CHT (Fig. 1). The demographic characteristics and outcomes of patients with TBI without ICH, and patients with ICH treated or not with CHT are described in Table 1. Continuous hyperosmolar therapy was initiated for a median duration of 5 (3–8) days.

**Table 1:** Comparison of patients with intracranial hypertension treated or not with continuous hyperosmolar therapy (CHT)

Characteristics	Without intracranial hypertension	With intracranial hypertension		
		Not treated with CHT	Treated with CHT	*P* values^a^
Number of patients	541	402	143	
Age, years	42 (25–58)	38 (24–53)	37 (21–57)	0.74
Male	430 (79.5)	323 (80.4)	114 (79.7)	0.87
Injury Severity Score	25 (16–34)	25 (17–33)	27 (25–41)	< 0.0001
Glasgow Coma Scale	6 (4–8)	5 (3–7)	6 (4–8)	0.0002
One or two non-reactive pupils, yes	98 (26)	102 (38.4)	45 (31.5)	0.17
Hypoxaemia, yes	62 (11.5)	79 (19.7)	41 (28.7)	0.03
Hypotension, yes	186 (34.4)	145 (36.1)	46 (32.2)	0.40
CT classification				0.002
I	20 (3.8)	11(2.8)	1 (0.7)	
II	174 (32.9)	94 (23.7)	43 (30.1)	
III	28 (5.3)	35 (8.8)	15 (10.5)	
IV	20 (3.8)	24 (6.1)	21 (14.7)	
V	169 (32.0)	165 (41.6)	40 (28.0)	
VI	118 (22.3)	68 (17.1)	23 (16.1)	
Corti-TC trial – inclusion, yes	161 (29.8)	134 (33.3)	31 (21.7)	0.009
Corti-TC trial – steroids, yes	85 (15.7)	63 (15.7)	16 (11.2)	0.191
BI-VILI trial – inclusion, yes	179 (33.1)	155 (38.6)	0 (0.0)	< .0001
BI-VILI trial – fully compliant, yes	1 (1.7)	0 (0.0)	0 (0.0)	-
Management of intracranial hypertension, *yes*				
Hyperosmolar therapy (bolus)	NA	309 (76.9)	82 (57.3)	< 0.0001
Barbiturate	NA	192 (47.8)	78 (54.6)	0.16
Hypothermia	NA	111 (27.6)	62 (43.4)	0.0005
Moderate hypocapnia	NA	61 (15.2)	5 (3.5)	0.0002
Decompressive craniectomy	NA	87 (21.6)	14 (9.8)	0.002
Duration of invasive ventilation, days	10 (5–19)	18 (12–27)	18 (13–26)	0.79*
ICU length of stay, days	15 (9–25)	24 (16–34)	25 (18–35)	0.93*
Decision to withdraw care in ICU, yes^b^	38 (10.0)	46 (17.2)	23 (16.1)	0.77
Survival				
In ICU	489 (90.4)	269 (66.9)	106 (74.1)	0.11
At day 90	475 (87.8)	265 (65.9)	106 (74.1)	0.07
GOS at day 90^c^				0.01
Dead	66 (17.0)	137 (42.6)	37 (26.1)	
Vegetative	6 (1.5)	10 (3.1)	5 (3.5)	
Severe disability	84 (21.6)	60 (18.6)	36 (25.4)	
Moderate disability	87 (22.4)	52 (16.2)	34 (23.9)	
Good recovery	146 (37.5)	63 (19.6)	30 (21.3)	
Moderate to good recovery (GOS 4–5)	133 (59.9)	115 (35.8)	64 (45.2)	0.06

Results express as median (25^th^–75^th^ percentile) or number (percentage)

Computed tomography (CT) classification: I, no visible intracranial pathology on CT scan; II, midline shift 0–5 mm; III, cisterns compressed or absent with midline shift 0–5 mm; IV, midline shift > 5 mm; V, any lesion surgically evacuated; VI, high-density or mixed-density lesion > 25 mm, not surgically evacuated

*ICU* Intensive Care Unit, *GOS* Glasgow outcome scale

^a^ *P* values for comparisons between patients with intracranial hypertension treated or not with continuous hyperosmolar therapy

^b^Available in 410 patients

^c^Available in 322 patients (80%) untreated with continuous hyperosmolar therapy and in 142 patients (99%) treated with continuous hyperosmolar therapy

*Log-rank test

At day 90, 475 patients (87.8%) without ICH were alive compared with 265 (65.9%) patients with ICH not treated with CHT (*p* < 0.001) and 106 patients (74.1%) with ICH and treated with CHT (*p* = 0.001).

### Effects of continuous hyperosmolar therapy in patients with traumatic brain injury with intracranial hypertension

**Fig. 2:**
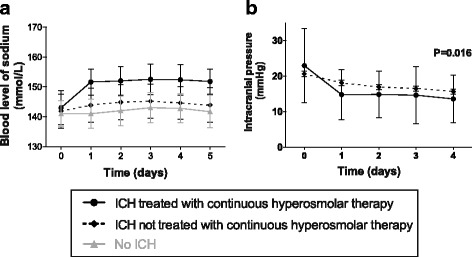
Time course of the blood levels of sodium (**a**) and of intracranial pressure (**b**) in patients treated or not with continuous hyperosmolar therapy. *ICH* intracranial hypertension

Blood levels of sodium were higher in patients with TBI with ICH than in patients without ICH (*p* < 0.001, Fig. 2a). Blood levels of sodium were higher in patients with TBI with ICH treated with CHT than in those not treated with CHT (*p* < 0.001, Fig. 2a). ICP levels were lower in patients with TBI with ICH treated with CHT than in those treated with standard treatment (Fig. 2b). Patients with TBI treated with CHT less frequently required the application of moderate hypocapnia (*p* = 0.0002) or decompressive craniectomy (*p* = 0.002) than patients whose care complied with the recommendations (Table 1).

### Primary outcome

**Fig. 3:**
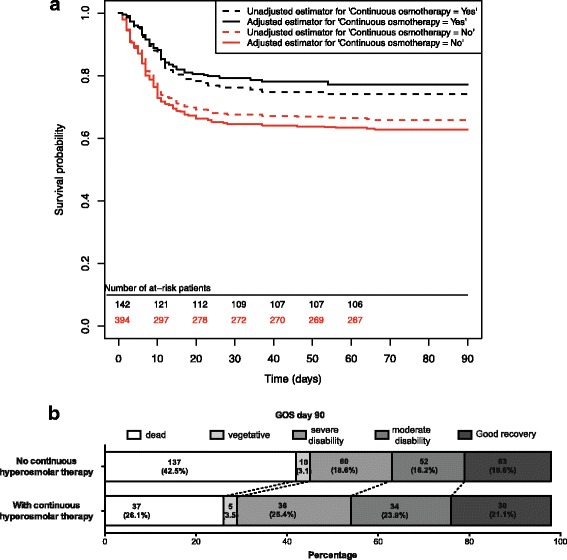
Comparison of 90-day outcomes in patients with traumatic brain injury (TBI) with intracranial hypertension (ICH) treated or not with continuous hyperosmolar therapy. **a** Cumulative incidence curves for survival at day 90. **b** Glasgow Outcome Scale (GOS) at day 90 in patients with TBI with ICH treated or not with continuous hyperosmolar therapy

In the population of patients with TBI with ICH, the crude HR for mortality at day 90 with CHT was 1.43 (95% CI, 0.99 - 2.06, *p* = 0.05, Fig. 3a). In propensity score analysis adjusted for predefined criteria (CT classification, age, GCS, non-reactive pupil, hypoxaemia and interventional study arms) [15] and baseline imbalances (Table 1), the adjusted HR for survival at day 90 was 1.74 (95% CI, 1.36 - 2.23, *p* < 0.001) (Fig. 3a). Sensitivity analysis to evaluate the robustness of this adjustment was performed, with multivariate analysis investigating the factors independently associated with survival at day 90 (Additional file 4: Table S1). In multivariate analysis, the adjusted HR for survival with early CHT was 1.98 (95% CI, 1.3 - 32.96, *p* < 0.001; Additional file 4: Table S1).

At day 90, the GOS distribution differed between the treated and untreated patients (*p* = 0.01, Fig. 3b). Favourable outcomes (pre-specified as moderate to good recovery on the GOS) occurred in 45.2% of patients with ICH treated with early CHT and in 35.8% of patients with ICH not treated with CHT (*p* = 0.06).

### Tolerance of continuous hyperosmolar therapy

The main side effect observed in patients treated with continuous hyperosmolar therapy was moderate hypernatraemia (145–159 mmol/L, Additional file 2: Figure S2A). Severe hypernatremia (≥ 160 mmol/L) was more frequent in treated patients (*n* = 13, 9.1%) vs. untreated patients (*n* = 9, 2.2%, *p* < 0.001). The time course of serum urea and of creatinine did not alter significantly during CHT (Additional file 5: Figure S3). No case of central pontine myelinolysis was recorded.

### Systematic review of the literature

Given the potential bias of this observational study, notably a potential centre-effect, the reported increase in the risk of survival associated with CHT could have been underestimated or overestimated. We therefore performed a review of the literature to compare our estimation of the effect of the treatment on survival with the effects that have been reported in previous studies.

**Fig. 4:**
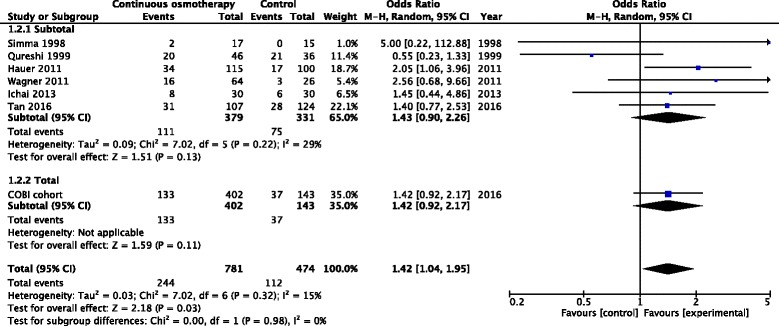
Number of patients deceased on day 28 or hospital discharge in the review of literature. *M-H* Mantel-Haenszel

Systematic review of the literature identified eight studies, including the current results that involved 1304 participants, with seven trials including 1255 participants providing in-hospital mortality data (Additional file 6: Figure S4). Descriptions of the studies are provided in Additional files 7 and 8: Tables S2 and S3. Mortality was less frequent in patients treated with CHT (intervention 112/474 (23.6%) vs. control 244/781 (31.2%); OR 1.42, 95% CI, 1.04–1.95), *p* = 0.03, *I*_2_ = 15%, Fig. 4). In sub-group analyses, the OR for survival with treatment changed little between the randomised clinical trials and the observational studies (OR 1.71 (95% CI, 0.55–5.26) vs. 1.39 (95% CI 0.95–2.05), respectively).

## Discussion

In this large prospective multicentre cohort, the early use of CHT in patients with TBI with ICH was safe and was independently associated with survival at day 90. Moreover, after incorporating the present results in a review of the literature, we confirmed that CHT was associated with decreased mortality. In the review of the literature, we identified low heterogeneity between the risk ratio (RR) for survival, suggesting that the effect of CHT is little altered by a centre-effect and could be extrapolated to daily practice.

ICH increases brain damage and its treatment remains critical for improving outcomes. The beneficial effects of hyperosmolar therapy have mainly been described for clinical variables such as control of ICP and for a few hours after administration of a bolus [8, 16, 17]. Our results show that ICP was lower and that surrogate markers of the course of ICH (boluses of hyperosmolar therapy, moderate hypocapnia or performance of craniectomy) are less frequently used in patients treated with CHT. When hyperventilation is recommended as a temporizing measure for the reduction of elevated ICP [6, 18], there are fewer reported episodes of hyperventilation therapy or hypothermia. This could also be considered as a beneficial effect of CHT. Given that we have previously reported no rebound of ICH during hyperosmolar therapy tapering [10], these results suggest that CHT provides prolonged control of ICP after acute brain injury. However, the protective effects observed with the use of CHT can be mediated by other mechanisms than ICP control [16, 19]. For example, CHT reduces the risk of hypovolaemia, which is associated with secondary brain injuries [20].

Before implementing CHT in clinical practice, it is critical to determine the timing of administration that will be the most efficient to enhance outcomes. In the COBI cohort study, we identified association between survival and treatment when CHT was used as a first-step treatment for ICH. Continuous hyperosmolar therapy has also been proposed as preventive treatment in brain-injured patients at risk of ICH [21–23]. Interestingly, our review of the literature provided evidence that CHT reduces the risk of ICH when applied as preventive therapy and we found little difference in the reduction of the risk of death between preventive and curative CHT. Taken altogether, these results suggest that CHT could be used early after TBI in patients deemed at high risk of developing ICH [21, 22, 24] or as a first-step treatment in patients developing ICH, and not only as a rescue therapy in the case of refractory ICH [10].

One of the main factors hindering the use of CHT is safety. Various neurologic complications including seizure, central pontine myelinolysis, and parenchymal accumulation of osmotic agents have been suspected. We did not record any neurological alterations that could be related to CHT, suggesting that it is well-tolerated. However, we are aware that potential clinical side effects could be missed in heavily sedated patients.

One of the main fears about the complications of CHT is hypernatraemia, because it has been associated with mortality [25, 26]. Hypernatraemia is a common complication after TBI, in up to 51.5% of patients, even without CHT, and it is currently impossible to know from these data if the increased risk of mortality described is related to an underlying medical condition or to hypernatraemia itself. It is of interest to note that only severe hypernatraemia (> 160 mmol/L) and not moderate hypernatraemia (145–159 mmol/L) is independently associated with mortality [27]. Close biological monitoring with the measurement of natraemia every 4–12 hours has been shown to enable control of natraemia within the ranges recommended by international guidelines (145–155 mmol/L, moderate hypernatremia) [18, 28] and to reduce the risk of severe hypernatraemia (Na^+^ > 160 mmol/L) in < 10% of patients treated with CHT [10, 21, 24]. Moreover, demonstration that poor tolerance of dysnatremia is mainly observed in the case of rapid variations in natraemia [29] provides a strong rationale to use a continuous infusion adapted to regular biological follow up rather than repeatable boluses of hyperosmolar therapy.

Alteration of renal function has also been reported during CHT [30]. Our results revealed no changes in urea or creatinine – suggesting no harm to the kidney. However, Froelich et al. reported a higher risk of pathological creatinine and urea levels in brain-injured patients receiving CHT without pre-specified biological monitoring [24]. Taken together, these data underline the need to set predetermined thresholds for natraemia, enabling a slow and controlled increase in natraemia along with close biological monitoring.

This study has several weaknesses. First, this observational cohort demonstrates an association but not a causal link between CHT and survival. Second, continuous osmotherapy was performed in one centre, exposing our results to a centre effect. Even if we found little heterogeneity in the estimation of the treatment effects between the studies included in the meta-analysis, suggesting that the effect of the treatment is robust to inter-centre variations in clinical practice, we cannot definitively rule out that other interventions participate in the better outcomes of patients treated with CHT. Notably, moderate hypocapnia and decompressive craniectomy were less frequently used in the group of patients treated than in patients not treated with CHT. Moreover, the blood electrolyte levels were probably more frequently measured in the CHT group than in the control group. A protocol of care with frequent electrolyte measures could enhance the outcomes of critically ill patients, as severe dysnatraemia (> 160 mmol/L) is associated with death [25, 26, 29]. However, protocol-based control of natraemia was used in the control group, and no severe dysnatraemia was apparent in this group (see Fig. 2a). Third, only three randomised trials were available for the review of literature [22, 31, 32]. We have included five before/after studies or quasi-experimental studies because their study designs provide a good level of evidence and an accurate estimation of the effect of the intervention [33]. The results of the systematic review were not significantly changed when only the randomised clinical trials were included. Fourth, the COBI cohort included patients with moderate to severe TBI while other studies covered in the systemic review included patients with severe brain injury. However, the heterogeneity between the subgroups (other studies vs. COBI cohort) for the risk of death was low (*I*_2_ = 0%). Moreover, the international recommendations on the methodology of clinical trials, which aimed to improve the power of neuro-reanimation trials, argue for the use of inclusion criteria that are as broad as possible, as long as they are compatible with the mechanisms of action of the evaluated intervention [34]. Since the secondary occurrence of ICH cannot be excluded in patients with moderate head trauma [35], patients with moderate to severe head trauma were included in this study. Fifth, the rate of pupillary abnormalities was higher in the COBI cohort than in recent studies in patients with moderate to severe TBI [36]. The COBI cohort included patients with moderate to severe TBI requiring mechanical ventilation, for whom the GCS scores were frequently quoted early at the trauma scene, and CHT was administered only in patients with ICH. We therefore cannot exclude that the recorded GCS score underestimates trauma severity and patients with moderate TBI were kept in the analysis. Moreover, CHT was administrated in patients with moderate TBI only in the case of secondary neurologic deterioration and ICH. Finally, and despite the very low incidence of side effects recorded in the present results, it should be noted that our study was not powered for a description of side effects.

## Conclusions

In conclusion, in this large multicentre cohort study, the use of CHT as a first-tier treatment for ICH was associated with the increased survival of patients with TBI. This association was confirmed in a systematic review including all available clinical studies. The risk of severe hypernatraemia during treatment justifies the setting of thresholds for acceptable hypernatraemia and adapting the flow of hyperosmolar therapy to close biological monitoring. As advocated by many other authors [21, 24, 37] and international guidelines [6], a randomised clinical trial appears to be urgently needed to confirm the effects of CHT on the outcomes of patients with TBI. To adequately address this issue, we designed the COBI study (Continuous hyperosmolar therapy for traumatic brain-injured patients, a multicentre randomised open-label trial with blinded adjudication of primary outcome – NCT03143751), which is powered to investigate the effects of CHT on the neurological outcomes as assessed by the GOS-Extended at 6 months [38].

## Additional files

Additional file 1: Figure S1. Stages of therapeutic management. (PDF 20 kb)

Additional file 2: Figure S2. Continuous hypertonic saline therapy. (PDF 20 kb)

Additional file 3: Supplementary methods for the review of literature. (PDF 184 kb)

Additional file 4: Table S1. Univariate and multivariate analysis for the risk factors of mortality at day 90 in patients with TBI with intracranial hypertension. (DOC 55 kb)

Additional file 5: Figure S3. Time course of the blood levels of creatinine (**A**) and urea (**B**) in patients treated or not with continuous hyperosmolar therapy. (PDF 67 kb)

Additional file 6: Figure S4. Flow chart of the literature research for the systematic review of literature. (PDF 46 kb)

Additional file 7: Table S2. Detailed characteristics of eligible studies. (DOC 53 kb)

Additional file 8: Table S3. Quality assessment of eligible studies. (DOC 49 kb)

## Abbreviations

CHT: Continuous hyperosmolar therapy
CT: Computed tomography
GCS: Glasgow Coma Score
GOS: Glasgow Outcome Scale
ICH: Intracranial hypertension
ICP: Intracranial pressure
ICU: Intensive care units
OR: Odds ratio
RR: Risk ratio
TBI: Traumatic brain-injury
