# Supplementary material for: Association between continuous hyperosmolar therapy and survival in patients with traumatic brain injury – a multicentre prospective cohort study and systematic review
Source: Crit Care. 2017 Dec 28;21:328. doi: 10.1186/s13054-017-1918-4 (PMC5745762; doi:10.1186/s13054-017-1918-4)
Supplement: Supplementary file 1 — Stages of therapeutic management. (PDF 20 kb) [file 13054_2017_1918_MOESM1_ESM.pdf]

Stage 1 for all comatose brain-injured patients

Sedation (intravenous)

Mechanical ventilation to

- ensure normocapnia
- prevent hypoxemia

Body temperature control (36-37°C)

Normoglycemia

Intracranial hypertension

Not treated with Continuous Hyperosmolar Therapy

Stage 2

Boluses of hyperosmolar therapy Mannitol 0.25 to 1 mg/kg of body weight

Or hypertonic saline solution, 250 mOsm dose

(blood osmolality < 320 mmol/L)

Treated with Continuous Hyperosmolar Therapy

Stage 2

CHT with NaCl20% (1-hr bolus followed by a continuous infusion)

+/- Boluses of hyperosmolar therapy

(blood osmolality < 340 mmol/L)

Continued Stages 1 and 2 as required

Stage 3 options for the treatment of refractory ICH (if required)

Barbiturate therapy

Therapeutic hypothermia (32-35°C)

Moderate hypocapnia (32-36 mmHg)

Decompressive Craniectomy
