# Supplementary material for: Association between continuous hyperosmolar therapy and survival in patients with traumatic brain injury – a multicentre prospective cohort study and systematic review
Source: Crit Care. 2017 Dec 28;21:328. doi: 10.1186/s13054-017-1918-4 (PMC5745762; doi:10.1186/s13054-017-1918-4)
Supplement: Supplementary file 2 — Continuous hypertonic saline therapy. (PDF 20 kb) [file 13054_2017_1918_MOESM2_ESM.pdf]

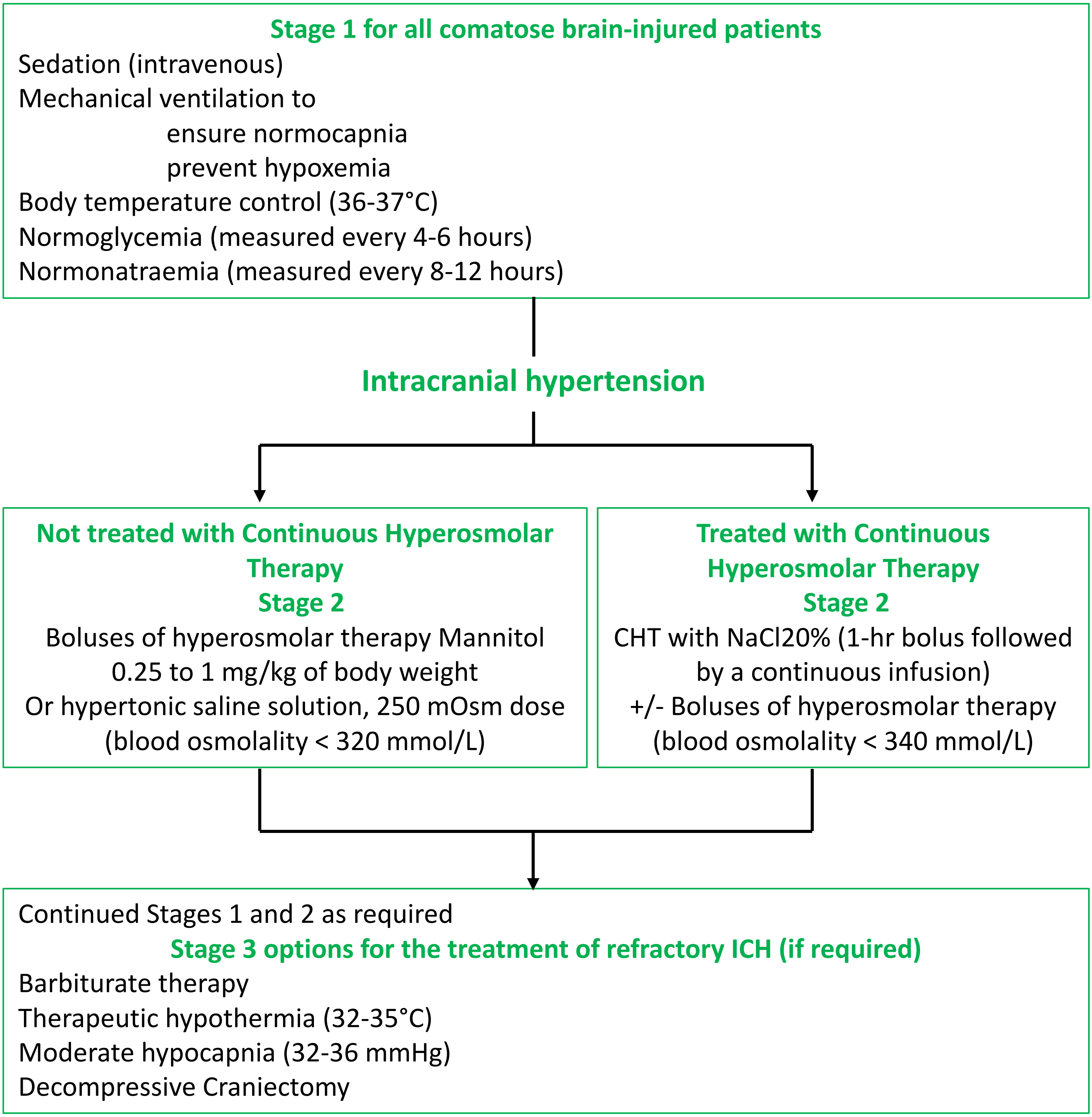
Stage 1 for all comatose brain-injured patients
Sedation (intravenous)
Mechanical ventilation to
ensure normocapnia
prevent hypoxemia
Body temperature control (36-37°C)
Normoglycemia (measured every 4-6 hours)
Normonatraemia (measured every 8-12 hours)
Intracranial hypertension
Not treated with Continuous Hyperosmolar Therapy
Stage 2
Boluses of hyperosmolar therapy Mannitol 0.25 to 1 mg/kg of body weight
Or hypertonic saline solution, 250 mOsm dose
(blood osmolality < 320 mmol/L)
Treated with Continuous Hyperosmolar Therapy
Stage 2
CHT with NaCl20% (1-hr bolus followed by a continuous infusion)
+/- Boluses of hyperosmolar therapy
(blood osmolality < 340 mmol/L)
Continued Stages 1 and 2 as required
Stage 3 options for the treatment of refractory ICH (if required)
Barbiturate therapy
Therapeutic hypothermia (32-35°C)
Moderate hypocapnia (32-36 mmHg)
Decompressive Craniectomy
