# Supplementary material for: Association between continuous hyperosmolar therapy and survival in patients with traumatic brain injury – a multicentre prospective cohort study and systematic review
Source: Crit Care. 2017 Dec 28;21:328. doi: 10.1186/s13054-017-1918-4 (PMC5745762; doi:10.1186/s13054-017-1918-4)
Supplement: Supplementary file 3 — Supplementary methods for the review of literature. (PDF 184 kb) [file 13054_2017_1918_MOESM3_ESM.pdf]

MOOSE Guidelines for Meta-Analyses and Systematic Reviews of Observational Studies*

Title

Identify the study as a meta-analysis (or systematic review) **page 1**

Abstract

Use the journal's structured format **page 3**

Introduction

Present

- The clinical problem **page 4**
- The hypothesis **page 4**
- A statement of objectives that includes the study population, the condition of interest, the exposure or intervention, and the outcome(s) considered **pages 4-5**

Sources

Describe

- Qualifications of searchers (eg, librarians and investigators) **supplemental material**
- Search strategy, including time period included in the synthesis and keywords
- Effort to include all available studies, including contact with authors
- Databases and registries searched **page 9**
- Search software used, name and version, including special features used (eg, explosion) **page 9**
- Use of hand searching (eg, reference lists of obtained articles) **supplemental material**
- List of citations located and those excluded, including justification
- Method of addressing articles published in languages other than English **page 9**
- Method of handling abstracts and unpublished studies **supplemental material**
- Description of any contact with authors **supplemental material**

Study Selection

Describe

- Types of study designs considered **page 9**
- Relevance or appropriateness of studies gathered for assessing the hypothesis to be tested **page 9**
- Rationale for the selection and coding of data (eg, sound clinical principles or convenience)
- Documentation of how data were classified and coded (eg, multiple raters, blinding, and interrater reliability) **supplemental material**
- Assessment of confounding (eg, comparability of cases and controls in studies where appropriate)
- Assessment of study quality, including blinding of quality assessors; stratification or regression on possible predictors of study results **supplemental material**
- Assessment of heterogeneity **supplemental material**
- Statistical methods (eg, complete description of fixed or random effects models, justification of whether the chosen models account for predictors of study results, dose-response models, or cumulative meta-analysis) in sufficient detail to be replicated **page 9**

Results

Present

- A graph summarizing individual study estimates and the overall estimate **Figure 4**
- A table giving descriptive information for each included study **Tables S2-S3**
- Results of sensitivity testing (eg, subgroup analysis) **page 12**
- Indication of statistical uncertainty of findings

Discussion

Discuss

- Strengths and weaknesses **page 15**
- Potential biases in the review process (eg, publication bias) **page 15**
- Justification for exclusion (eg, exclusion of non–English-language citations)
- Assessment of quality of included studies **table S3**
- Consideration of alternative explanations for observed results **page 15**
- Generalization of the conclusions (ie, appropriate for the data presented and within the domain of the literature review) **page 16**
- Guidelines for future research **page 16**
- Disclosure of funding source **page 17**

*Modified from Stroup DF, Berlin JA, Morton SC, Olkin I, Williamson GD, Rennie D, et al. Meta-analysis of observational studies in epidemiology: a proposal for reporting. Meta-analysis Of Observational Studies in Epidemiology (MOOSE) group. JAMA 2000;283:2008–12. Copyrighted © 2000, American Medical Association. All rights reserved.
