# Supplementary material for: Association between continuous hyperosmolar therapy and survival in patients with traumatic brain injury – a multicentre prospective cohort study and systematic review
Source: Crit Care. 2017 Dec 28;21:328. doi: 10.1186/s13054-017-1918-4 (PMC5745762; doi:10.1186/s13054-017-1918-4)
Supplement: Supplementary file 4 — Univariate and multivariate analysis for the risk factors of mortality at day 90 in patients with TBI with intracranial hypertension. (DOC 55 kb) [file 13054_2017_1918_MOESM4_ESM.doc]

**Early continuous hyperosmolar therapy for intracranial hypertension after traumatic brain injury**

The COBI multicenter prospective cohort study and systematic review

**Table S1 – Univariate and multivariate analysis for the risk factors of mortality at day 90 in TBI patients with intracranial hypertension**

|  | **Survival at day 90** | | **Risk factors of survival at day 90** | | | |
| --- | --- | --- | --- | --- | --- | --- |
|  | **Survivors** | **Non-survivors** | **Univariate**  **HR (95%CI)** | **P**  **values** | **Multivariate HR (95%CI)** | **P values** |
| Number of patients | 371 | 174 |  |  |  |  |
| Age, *years* | 36.3 ± 16.3 | 47.3 ± 18.2 | 0.97 (0.96-0.98) | <0.001 | 0.97 (0.96-0.98) | <0.001 |
| Male | 295 (79.5) | 142 (81.6) | 0.90 (0.61-1.32) | 0.584 | NS | NS |
| Injury Severity Score | 28.2 ± 12.3 | 28.7 ± 13.6 | 1.00 (0.98-1.01) | 0.617 | NS | NS |
| Glasgow Coma Scale | 6 (4-8) | 4 (3-7) | 1.17 (1.10-1.24) | <0.001 | 1.14 (1.07-1.21) | <0.001 |
| 1 or 2 non-reactive pupil*, yes* | 84 (31.5) | 63 (44.4) | 0.61 (0.44-0.85) | 0.004 | 0.68 (0.48-0.95) | 0.026 |
| Hypoxemia, *yes* | 83 (22.4) | 37 (21.2) | 1.15 (0.80-1.65) | 0.456 | 1.61 (1.08-2.42) | 0.021 |
| Hypotension, *yes* | 133 (35.9) | 58 (33.3) | 1.09 (0.80-1.50) | 0.586 | NS | NS |
| CT classification | 5 (2-5) | 5 (3-5) | 0.96 (0.86-1.05) | 0.358 | 1.01 (0.89-1.13) | 0.877 |
| Corti-TC trial – Inclusion, *yes*  Corti-TC trial – Steroids, *yes*  BI-VILI trial – Inclusion, *yes*  BI-VILI trial – Fully compliant, *yes* | 126 (34.0)  59 (15.9)  99 (26.7)  0 (0.0) | 39 (22.4)  20 (11.5)  56 (32.2)  0 (0.0) | 1.68 (1.17-2.40)  1.41 (0.89-2.29)  0.82 (0.59-1.12)  - | 0.005  0.148  0.212  - | NS  0.81 (0.28-2.31)  NS  - | NS  0.691  NS  - |
| Decision of care withdrawal, *yes* | 9 (3.4) | 60 (41.7) | 0.20 (0.14-0.28) | <0.001 | 0.28 (0.19-0.40) | <0.001 |
| Management of intracranial hypertension, *yes*  Osmotherapy (bolus)  Continuous hyperosmolar therapy  Barbiturate  Hypothermia  Moderate hypocapnia  Decompressive craniectomy | 251 (67.7)  106 (28.6)  165 (44.5)  105 (28.3)  37 (10.0)  78 (21.0) | 140 (80.5)  37 (21.3)  105 (60.3)  68 (39.1)  29 (16.7)  23 (13.2) | 0.56 (0.38-0.81)  1.43 (0.99-2.06)  0.59 (0.44-0.80)  0.69 (0.51-0.94)  0.59 (0.40-0.88)  1.59 (1.02-2.46) | 0.002  0.052  <0.001  0.017  0.009  0.039 | NS  1.98 (1.33-2.96)  0.47 (0.33-0.67)  NS  NS  1.90 (1.15-3.14) | NS  <0.001  <0.001  NS  NS  0.013 |

ICU Intensive Care Unit

Results express as mean ± standard deviation or median (25^th^-75^th^ percentile) or N (%)

NS: Non significate
