# Supplementary figures and images for: Association between continuous hyperosmolar therapy and survival in patients with traumatic brain injury – a multicentre prospective cohort study and systematic review
Source: Crit Care. 2017 Dec 28;21:328. doi: 10.1186/s13054-017-1918-4 (PMC5745762; doi:10.1186/s13054-017-1918-4)

Figure S3

A.

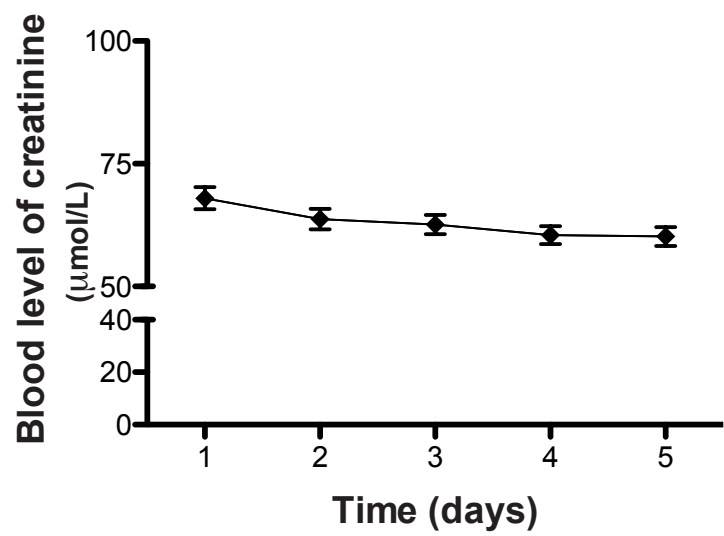

B.

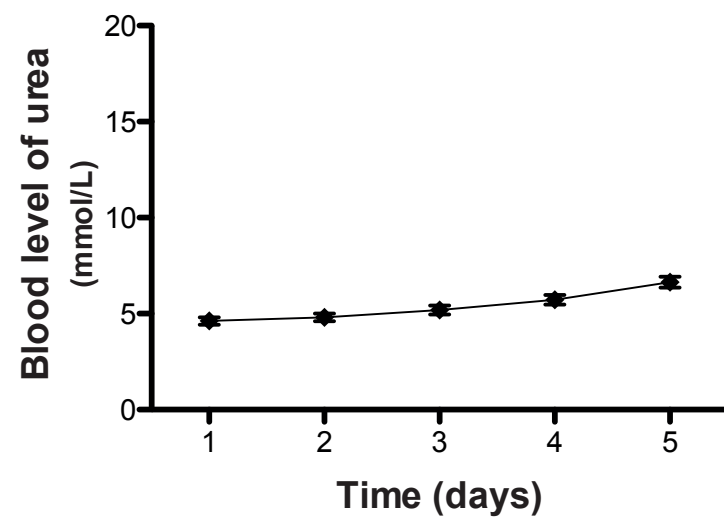

Supplement: Supplementary file 5 — Time course of the blood levels of creatinine (A) and urea (B) in patients treated or not with continuous hyperosmolar therapy. (PDF 67 kb) [file 13054_2017_1918_MOESM5_ESM.pdf]

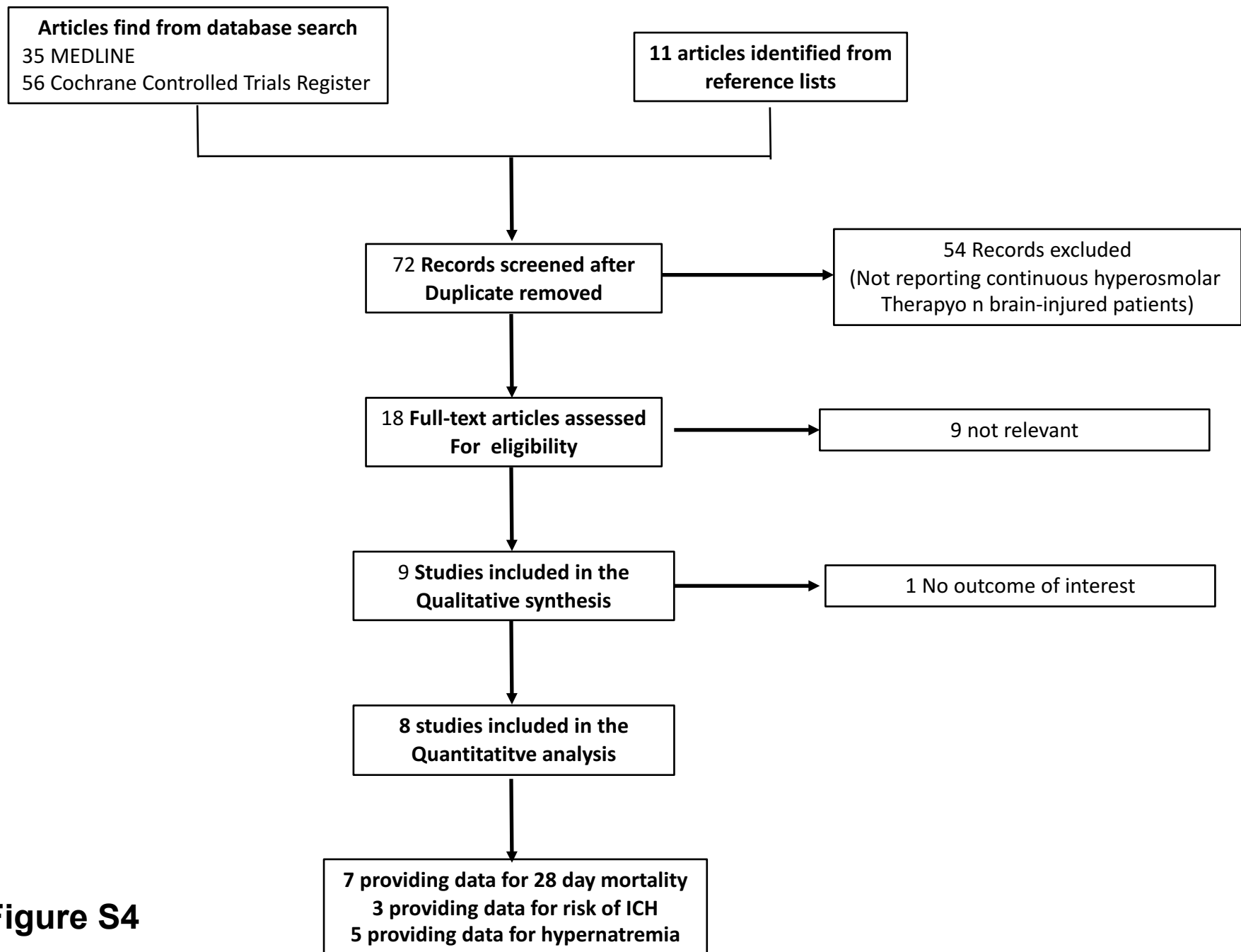

**Figure S4**

Supplement: Supplementary file 6 — Flow chart of the literature research for the systematic review of literature. (PDF 46 kb) [file 13054_2017_1918_MOESM6_ESM.pdf]
