# Supplementary material for: Association between continuous hyperosmolar therapy and survival in patients with traumatic brain injury – a multicentre prospective cohort study and systematic review
Source: Crit Care. 2017 Dec 28;21:328. doi: 10.1186/s13054-017-1918-4 (PMC5745762; doi:10.1186/s13054-017-1918-4)
Supplement: Supplementary file 7 — Detailed characteristics of eligible studies. (DOC 53 kb) [file 13054_2017_1918_MOESM7_ESM.doc]

**Early continuous hyperosmolar therapy for intracranial hypertension after traumatic brain injury**

The COBI multicenter prospective cohort study and systematic review

**Table S2: Detailed Characteristics of Eligible Studies**

| **First author** | **Year** | **Studied population (TBI, SAH, comatose)** | **Inclusion criteria**  **Glasgow Coma scale** | **Intervention (hypertonic NaCl or half-molar sodium lactate)** | **Preventive (before ICH) or curatige (after the first episode of ICH)** | **Number of patients treated with continuous osmotherapy** | **Number of patients not treated with continuous osmotherapy** |
| --- | --- | --- | --- | --- | --- | --- | --- |
| HAUER | 2011 | SAH | 3-12 | Hypertonic NaCl 3% | Preventive | 100 | 34 |
| ICHAI | 2013 | TBI | <9 | half molar sodium lactate | Preventive | 30 | 30 |
| WAGNER | 2011 | SAH | <9 | Hypertonic NaCl 3% | Preventive | 26 | 64 |
| FROELICH | 2009 | Comatose | <9 | Hypertonic NaCl 3% | Curative | 107 | 80 |
| SIMMA | 1998 | TBI | <9 | Hypertonic NaCl 1.6% | Preventive | 18 | 16 |
| TAN | 2016 | TBI | <9 | Hypertonic NaCl 3% | Curative | 124 | 107 |
| QURESHI | 1999 | TBI |  | Acetate Hypertonic 2-3% | Preventive | 36 | 46 |
| COBI Cohort | 2016 | TBI |  | Hypertonic NaCl 20% | Curative | 143 | 402 |

TBI: traumatic brain injury, SAH: subarachnoid hemorrhage, ICH: intracranial hypertension,
