# Supplementary material for: Association between continuous hyperosmolar therapy and survival in patients with traumatic brain injury – a multicentre prospective cohort study and systematic review
Source: Crit Care. 2017 Dec 28;21:328. doi: 10.1186/s13054-017-1918-4 (PMC5745762; doi:10.1186/s13054-017-1918-4)
Supplement: Supplementary file 8 — Quality assessment of eligible studies. (DOC 49 kb) [file 13054_2017_1918_MOESM8_ESM.doc]

**Early continuous hyperosmolar therapy for intracranial hypertension after traumatic brain injury**

The COBI multicenter prospective cohort study and systematic review

**Table S3. Quality Assessment of Eligible Studies**

|  |  | **Selection bias** | | **Performance bias** | **Detection bias** | **Attrition bias** | **Reporting bias** | **Other bias** |
| --- | --- | --- | --- | --- | --- | --- | --- | --- |
| **First author** | **year** | **Random sequence generation** | **Allocation concealment** | **Blinding of participants and personnel** | **Blinding of outcome assessment** | **Incomplete outcome data** | **Selective reporting** | **Other bias** |
| HAUER | 2011 | **-** | **-** | **-** | **-** | **+** | **+** | **-** |
| ICHAI | 2013 | **+** | **+** | **+** | **+** | **+** | **-** | **+** |
| WAGNER | 2011 | **-** | **-** | **-** | **-** | **+** | **+** | **-** |
| FROELICH | 2011 | **-** | **-** | **-** | **-** | **-** | **+** | **-** |
| SIMMA | 1998 | **+** | **+** | **+** | **+** | **-** | **+** | **+** |
| TAN | 2016 | **-** | **-** | **-** | **-** | **+** | **+** | **+** |
| QURESHI | 1999 | **-** | **-** | **-** | **-** | **+** | **+** | **-** |
| COBI Cohort | 2016 | **-** | **-** | **-** | **-** | **+** | **+** | **+** |

+ low risk of bias, ? Unclear risk of bias, - high risk of bias
